# Periocular skin warming promotes body heat loss and sleep onset: a randomized placebo-controlled study

**DOI:** 10.1038/s41598-020-77192-x

**Published:** 2020-11-23

**Authors:** Tomohisa Ichiba, Masahiro Suzuki, Sayaka Aritake-Okada, Makoto Uchiyama

**Affiliations:** 1 Personal Health Care Laboratory, Kao Corporation, 2-1-3, Bunka, Sumida-ku, Tokyo, 131-8501 Japan; 2 Department of Psychiatry, Nihon University School of Medicine, 30-1 Oyaguchi Kamicho, Itabashi-ku, Tokyo, 173-8610 Japan; 3 Faculty of Health and Social Services, Saitama Prefectural University, 820, Sannomiya, Koshigaya, Saitama 343-8540 Japan; 4 Tokyo Adachi Hospital, 5-23-20 Hokima, Adachi-ku, Tokyo, 121-0064 Japan

**Keywords:** Neurological disorders, Neuronal physiology, Autonomic nervous system

## Abstract

Periocular skin warming was reported to have favorable effects on subjective and objective sleep quality. We hypothesized that enhancing body heat loss by periocular skin warming would reduce sleep onset and improve sleep quality. Eighteen healthy volunteers were asked to maintain wakefulness with their eyes closed for 60 min after applying either a warming or sham eye mask, followed by a 60-min sleep period. Compared to the sham, periocular warming increased the distal skin temperature and distal–proximal skin temperature gradient only during the 30-min thermal manipulation period. In the subsequent sleep period, periocular warming facilitated sleep onset, increased stage 2 sleep and electroencephalographic delta activity during the first half of the sleep period relative to the sham. These results suggest that periocular skin warming may accelerate and deepen sleep by enhancing physiological heat loss via the distal skin, mimicking physiological conditions preceding habitual sleep.

## Introduction

Habitual sleep onset is usually preceded by a decline in core body temperature (CBT)^1^. The decline in CBT is promoted by the enhancement of heat loss which results in an increase in the distal skin temperature related with distal skin vasodilation^1–4^. Kräuchi and colleagues have reported that heat loss could be indirectly measured by assessing the temperature gradient from the proximal (i.e., infraclavicular, thigh, and stomach) skin to the distal (i.e., foot and hand) skin, known as the distal–proximal skin temperature gradient (DPG), and that the DPG was a good predictor of sleepiness and correlated with sleep onset latency (SOL)^3,5,6^. Furthermore, they have documented that cold feet or hands due to vasoconstriction syndrome have been associated with sleep onset difficulties^7–10^, indicating that the body’s physiological state was not yet ready for sleep due to insufficient heat loss caused by the impaired distal vasodilation^3,7–10^. Thus, changes in skin temperature play an important role in sleep regulation.

Many human studies have investigated that the effects of whole or localized body temperature manipulation on sleep regulation, by applying a warm bath^11–14^, a high heat capacity mattress^15^, air conditioning^16^, bed socks^17^, or a thermosuit^18,19^. Some studies have demonstrated that whole body warming by a warm bath 1–2 h before bedtime reduced SOL and increased slow-wave sleep^11–14^. Furthermore, regarding the local warming of the body, a study has demonstrated that foot temperature manipulation with bed socks was associated with reduced SOL in young and elderly subjects^17^. These findings in which sleep was successfully induced after thermal manipulation, may be interpreted as a consequence of the heat loss associated with the elevation of the distal skin temperature.

We have previously developed a disposable heat- and steam generating sheet (HSG-sheet) which can safely and easily manipulate the local skin temperature (e.g., the periocular^20–24^, neck^23^, and abdominal^25^ skin region) at home. Our studies has reported that periocular skin warming before bedtime improved the subjective sleep quality^22,23^ and increased the electroencephalography (EEG) delta power^23^ during the first half of the sleep episode in adults with mild difficulty falling asleep. Recently, we have demonstrated that periocular skin warming increased the distal skin temperature in the hand and foot, which indicated enhancement of the physiological heat loss process^24^. However, it is unclear whether periocular skin warming modulate sleep through physiological heat loss.

In the present study, to clarify the effect of periocular skin warming on sleep, we used a previously developed eye mask with HSG sheets and measured the skin and core body temperature as well as EEG during the sleep period under controlled experimental conditions. This minimizes the confounding effects of posture, food intake, and ambient temperature, as described in a previous study^24^. We then evaluated the changes in the distal and proximal skin and core body temperatures, as well as in sleep after periocular warming.

## Results

### Subjects

Two of twenty health subjects were excluded due to the impairment of the device during the experimental period. Finally, the data of eighteen male subjects (aged 29–57 years, mean ± SD: 45.4 ± 7.7 years) were included in the analysis.

### Body temperature, heart rate, and subjective sleepiness

**Figure 1:**
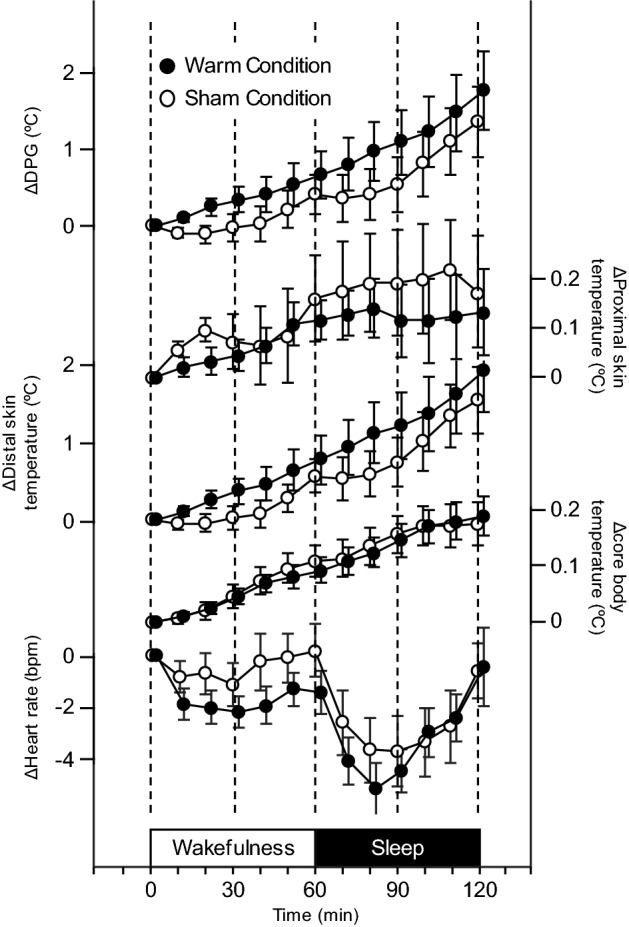
Time course (10 min means) of distal–proximal skin temperature gradient (DPG), proximal skin (infraclavicular) temperature, distal skin (foot) temperature, core body (rectum) temperature, and heart rate during the thermal manipulation period, observation period, and sleep period under the warming condition (closed circle) and sham condition (open circle). All values are presented as mean ± SEM of eighteen subjects.

**Figure 2:**
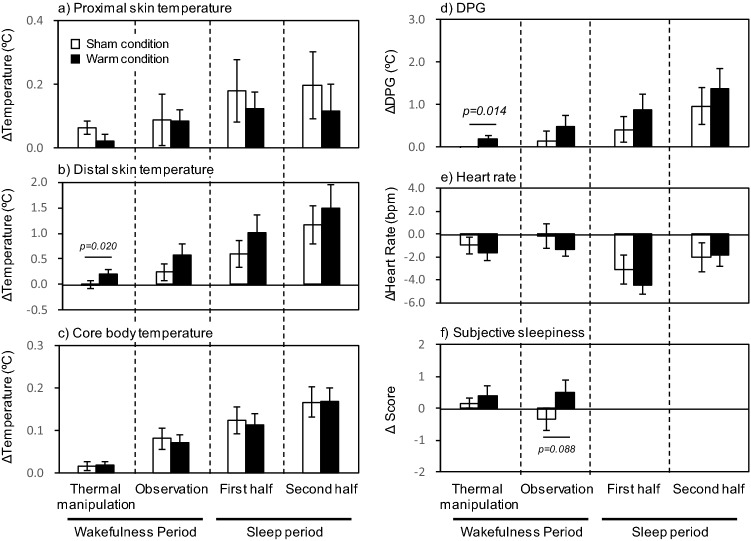
(**a**) Proximal skin (infraclavicular) temperature, (**b**) distal skin (foot) temperature, (**c**) core body (rectum) temperature, (**d**) distal–proximal skin temperature gradient (DPG), (**e**) heart rate, and (**f**) subjective sleepiness score during the thermal manipulation period, the observation period, and the first and second half of the sleep periods (respectively, 30 min means) under the warming and sham conditions. All values are expressed as mean ± SEM of eighteen subjects. Comparisons were performed relative to the sham condition using the Wilcoxon signed rank test.

The body temperature measures, heat rate, and subjective sleepiness score obtained just before the thermal manipulation period did not differ between the sham and warming conditions (Table 1). Figure 1 represents changes in the body temperature and heart rate throughout the experimental period. During the 30-min thermal manipulation period in which the periocular skin temperature was manipulated by a warming or sham eye mask, the foot skin temperature significantly increased under the warming condition compared with the sham condition (Fig. 2b), while the infraclavicular and rectum temperature did not differ between the two conditions (Fig. 2a,c). Similarly, the hand skin temperature during the thermal manipulation period significantly increased under the warming condition compared with the sham condition (p = 0.012). The DPG during the thermal manipulation period was significantly higher under the warming condition than under the sham condition (Fig. 2d), while no differences in the DPG were observed between the sham and warming conditions during the subsequent observation and sleep periods. The heart rate did not differ between the sham and warming conditions throughout the experimental period (Fig. 2e). Although the subjective sleepiness scores did not significantly differ between the sham and warming conditions, there was a trend for enforced subjective sleepiness under the warming condition compared with the sham condition during the observation period (p = 0.088; Fig. 2f).

**Table 1:** Baseline body temperature, heart rate, and subjective sleepiness scale.

	Sham	Warm	*p-Value*
Proximal skin temperature (ºC)	34.65 ± 0.13	34.69 ± 0.13	0.845
Distal skin temperature (ºC)	29.33 ± 0.51	29.61 ± 0.42	0.557
Core body temperature (ºC)	36.79 ± 0.06	36.75 ± 0.08	0.528
DPG (ºC)	-5.32 ± 0.55	-5.08 ± 0.47	0.557
Heart rate(bpm)	67.77 ± 2.05	68.43 ± 1.47	0.557
Subjective Sleepiness Scale	6.6 ± 0.3	6.2 ± 0.3	0.353

All are values expressed as mean ± SEM of eighteen subjects. Comparisons were performed relative to the sham condition using the Wilcoxon signed rank test.

DPG, distal–proximal skin temperature gradient.

### Sleep parameters

**Figure 3:**
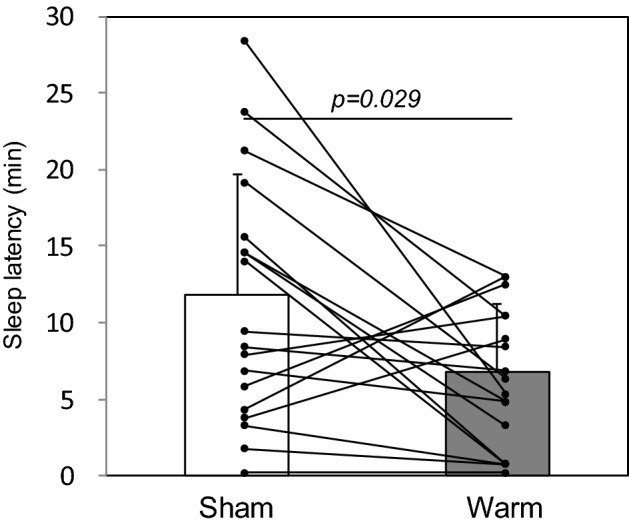
Sleep onset latency (SOL) between the sham and warming conditions. All values are expressed as mean ± SEM of 18 subjects. The scatterplots of the individual SOL are shown. Comparisons were performed relative to the sham condition using the Wilcoxon signed rank test.

SOL was significantly shortened in the warming condition compared to the sham condition (p = 0.029; Table 2 and Fig. 3). Other sleep parameters did not differ between the sham and warming conditions. To observe the temporal features of the effects, the 60-min sleep period was divided into two: the first half and the second half (Table 3). During the first half of the sleep period, stage 2 sleep was significantly increased under the warming condition compared to the sham condition (p = 0.029; Table 3).

**Table 2:** EEG sleep parameters during the whole sleep period.

	Sham	Warm	*P *value
SOL (min)	12.9 ± 2.3	6.8 ± 1.0	0.029
Stage 1 (min)	13.4 ± 2.2	10.0 ± 1.4	0.368
Stage 2 (min)	11.1 ± 2.5	13.4 ± 2.6	0.523
Stage 3 (min)	2.0 ± 0.7	1.1 ± 0.5	0.350
REM (min)	3.6 ± 1.4	4.0 ± 1.5	0.582
Wakefulness (min)	30.4 ± 3.7	30.8 ± 3.9	0.862
WASO (min)	9.6 ± 2.3	12.6 ± 2.2	0.184
TST (min)	29.9 ± 3.8	29.8 ± 3.9	0.913

All values are expressed as mean ± SEM of eighteen subjects. Comparisons were performed relative to the sham condition using the Wilcoxon signed rank test.

SOL, sleep onset latency; REM, rapid eye movement; WASO, wake after sleep onset; TST, total sleep time.

**Table 3:** EEG sleep parameters in first and second halves of the sleep period.

	First half of the sleep period			Second half of the sleep period		
	Sham	Warm	*P *value	Sham	Warm	*P *value
Stage1 (min)	6.0 ± 1.2	5.9 ± 0.7	0.571	5.3 ± 1.2	4.1 ± 1.0	0.058
Stage2 (min)	4.6 ± 1.2	8.4 ± 1.3	0.029	6.6 ± 1.6	5.8 ± 1.4	0.169
Stage3 (min)	0.5 ± 0.2	0.5 ± 0.3	0.905	2.5 ± 0.6	1.5 ± 0.4	0.260
REM (min)	3.6 ± 1.4	4.0 ± 1.5	0.582	2.5 ± 0.6	4.3 ± 1.0	0.634
Wakefulness (min)	16.7 ± 1.9	13.4 ± 1.8	0.064	9.8 ± 2.3	10.4 ± 2.5	0.155

All are values expressed as mean ± SEM of eighteen subjects. Comparisons were performed relative to the sham condition using the Wilcoxon signed rank test.

REM, rapid eye movement.

### Spectral analysis of EEG

**Figure 4:**
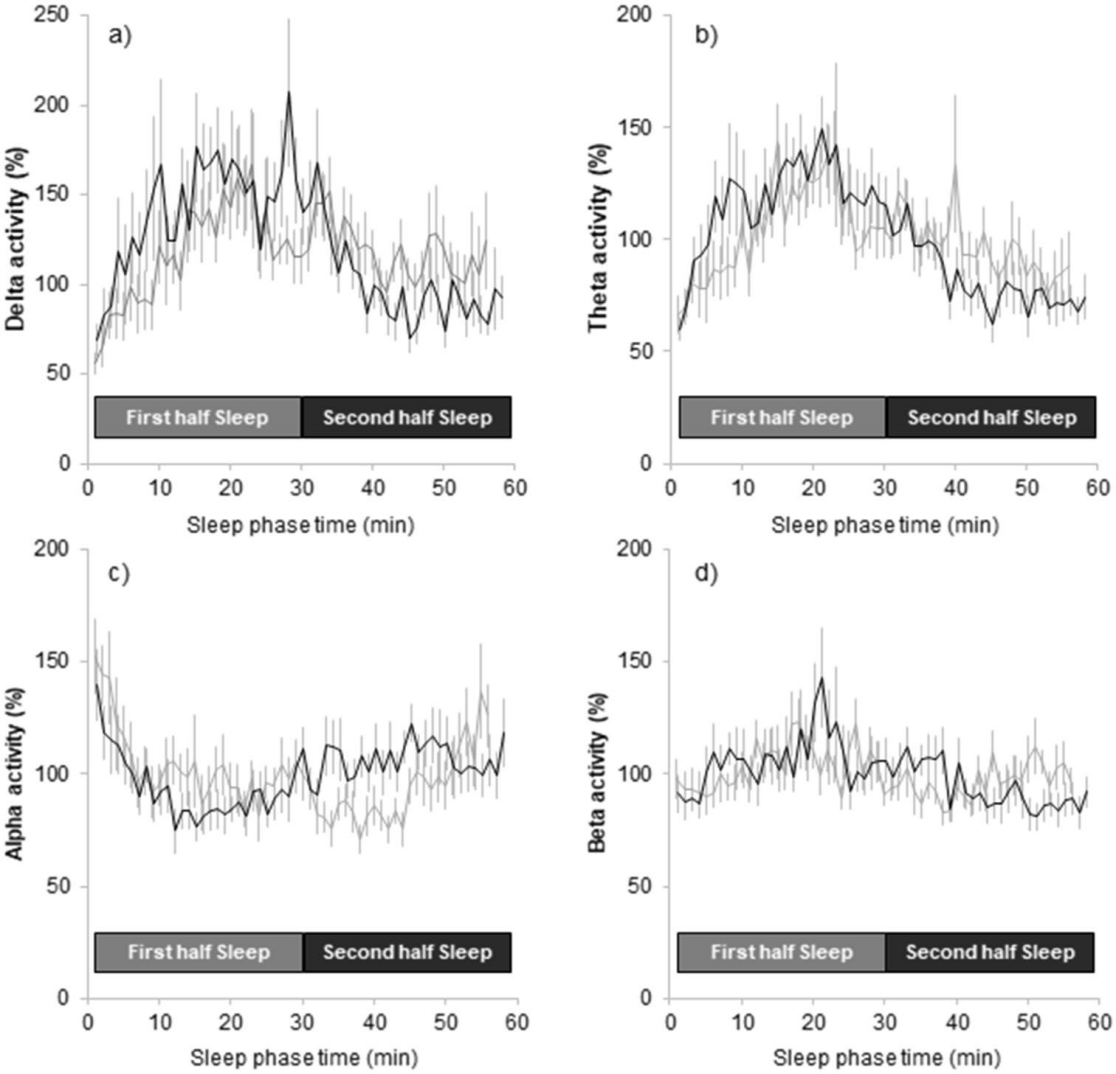
Time course of (**a**) delta activity (1.0–4.0 Hz), (**b**) theta activity (4.0–7.0 Hz), (**c**) alpha activity (8.0–12.0 Hz), and (**d**) beta activity (13.0–20.0 Hz) during the sleep session. Y axis represents the normalized values of each frequency based on the mean of each frequency during sleep session. All values are expressed as means ± SEM of eighteen subjects. The black line represents the warming condition, and the gray line represents the sham condition.

Figure 4 represents the time course of the EEG frequency activities (delta, theta, alpha, and beta activities). The EEG frequency activities during the 60-min sleep did not differ between the sham and warming conditions. Then, the EEG frequency activities were divided into the first and second halves of the 60-min sleep period (Table 4). Delta activity during the first half of the sleep period was significantly increased in the warming condition compared to the sham condition (p = 0.039), while the other frequency activities did not differ between the sham and warming conditions.

**Table 4:** Sleep EEG activity during the sleep phase.

	First half of the sleep period			Second half of the sleep period		
	Sham	Warm	*P *value	Sham	Warm	*P *value
Delta activity (%)	120.0 ± 7.1	137.6 ± 8.7	0.039	110.2 ± 12.8	88.5 ± 7.8	0.124
Theta activity (%)	104.6 ± 4.0	112.6 ± 3.2	0.064	88.9 ± 8.5	74.2 ± 6.5	0.113
Alpha activity (%)	99.2 ± 2.7	95.9 ± 2.3	0.286	102.7 ± 5.8	108.4 ± 4.7	0.407
Beta activity (%)	100.7 ± 1.7	105.4 ± 2.5	0.145	99.3 ± 3.5	88.8 ± 5.2	0.076

All values are expressed as means ± SEM of eighteen subjects. Comparisons were performed relative to the sham condition using the Wilcoxon signed rank test.

### Correlations between SOL and body temperatures

**Figure 5:**
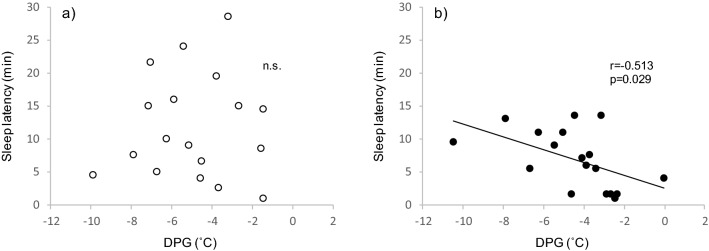
Correlation between sleep onset latency (SOL) and distal–proximal skin temperature gradient (DPG) in the (**a**) sham condition and (**b**) warming conditions. X axis represents the DPG just before the sleep period. n.s.; not significant.

There were no significant correlations with SOL and changes in foot skin temperature, CBT, or DPG in the sham and warming conditions. DPG prior to the sleep period was significantly and negatively correlated with SOL in the warming condition (r =  − 0.51, p = 0.029; Fig. 5). Similarly, the foot skin temperature prior to the sleep period significantly and negatively correlated with SOL (r =  − 0.50, p = 0.034). In the sham condition, however, there were no significant correlations for DPG (r = 0.04, p = 0.848; Fig. 5) and foot skin temperature (r = 0.01, p = 0.97) prior to the sleep period with SOL.

## Discussion

The present study was conducted using a semi-constant routine protocol in which posture, movement, food intake, and ambient temperature were controlled to clarify the effect of periocular skin warming on sleep. Periocular skin warming was revealed to increase the distal skin temperature and DPG, then enhance the subjective sleepiness, which subsequently shortened the SOL and increased stage 2 sleep for the first half of the sleep period. Using EEG spectral analysis, we also found that delta activity during the first half of the sleep period increased after periocular skin warming.

Based on previous studies demonstrating that thermal manipulation promoted sleep induction^11,17,18^, we hypothesized that periocular skin warming may have shortened SOL by accelerating heat loss, which is typically represented by an elevation in the distal skin temperature.

Periocular skin warming increased the distal skin temperature and DPG without affecting the proximal and core body temperatures, as previously reported^24^. The increases in the distal skin temperature and DPG, which were reportedly characteristic peripheral manifestations associated with sleep promoting process of the brain^1–4^, were observed only during the thermal manipulation period, not but during the observation and sleep periods.

Our results are comparable with our previous findings that periocular warming before bedtime improved subjective sleep initiation and enhanced delta power in the first 90-min period of the nocturnal sleep^23^. The results obtained through qualitive and quantitative EEG analyses in the present study clearly indicated that periocular skin warming accelerated and deepened non-rapid eye movement (NREM) sleep. Likewise, prior human studies found that homeostatic build-up of sleep pressure^26^ or pharmacological intervention^27^ led to the enhancement of sleep. Van Someren and colleagues have demonstrated that continuous skin temperature manipulation using a thermo-suit enhanced and deepened sleep^28^, and suggested that the effects may be due to modulation of brain areas involved in sleep regulation^29^.

In the present study, distal skin temperature elevation or heat loss enhanced by periocular skin warming during the thermal manipulation period disappeared during the subsequent periods, when sleep was robustly enhanced and deepened. Our findings can be interpreted as follows; periocular skin warming may have triggered brain mechanisms responsible for NREM sleep control, suggesting a novel interaction between thermal manipulation and sleep.

The effects of periocular skin warming on sleep enhancement might be interpreted by methods other than thermo regulation. Our prior study indicated that the sleep inducing effects of periocular skin warming were strongly related to psychological comfortableness^24^. Many prior studies have reported that less subjective anxiety at bedtime has been associated with reduced sleep latency and elevated deep sleep^30^. Our prior study documented that steam inhalation before bedtime first induced psychological relaxation and then increased delta power value^31^. The present results might be considered as a consequence of the psychological and physiological effects documented in such prior studies.

DPG prior to the sleep period showed significant correlation with SOL in the warming condition, while it was not correlated with SOL in the sham condition. There were no significant correlations between SOL and change in body temperature or DPG. Previously, changes in DPG have been demonstrated to significantly correlate with SOL in the administration of ramelteon^32^. We postulated that the 30-min observation period might have influenced the detection of association between sleep and changes in thermoregulatory measures.

Regarding the relationship between skin warming and sleep, the location on the skin, temperature, and timing of skin warming are likely to play an important role in sleep. A few studies have reported that the warming the foot skin accelerated sleep onset^17,33^, whereas others have showed no significant difference in SOL^34,35^. Additionally, Van Someren and colleague have demonstrated that proximal skin warming was more effective than distal skin warming^18,19^. However, it is not clear which location on the human skin is most effective to promote sleep. Previous^22,23^ studies and present study have showed that appropriate periocular skin warming has favorable effects on sleep. We postulate that the periocular skin region might be regarded as one closest regions of the eyes which is directly connected to the brain.

The present result that effects of periocular warming had favorable effects in inducing sleep may conversely indicate a possibility that appropriate periocular cooling could impede sleepiness and help in maintaining wakefulness because prior studies have suggested some thermoregulatory interventions to ameliorate pathologic hypersomnolence^36–38^.

This study had several limitations. First, the experimental studies were conducted in healthy male subjects aged 29–57 years. Difference in age has been reported to negatively affect thermoreception and temperature regulation^37^. Previously, Van Someren and colleague have demonstrated age-related changes in the thermoregulatory response during skin temperature manipulation^17^. Furthermore, this study did not include female subjects. Therefore, further study is required to clarify the effects of periocular warming on age- or sex-related thermoregulatory functions. Second, although our previous report suggested an improvement in subjective sleep initiation by periocular skin warming^23^, the present results alone may not sufficiently reveal the effects of periocular warming on nocturnal sleep because this study examined the effects of periocular warming only during the ascending portion of circadian core body temperature fluctuation, in the daytime. It is necessary to verify whether periocular skin warming has favorable effects on sleep during the night, when circadian core body temperature fluctuation shows descending trend. Further studies under nocturnal sleep conditions are required to confirm the direct relationship between periocular warming and nocturnal sleep. Third, there were no significant differences in the subjective sleepiness score between the sham and warming conditions in the present study. It has been reported that objective measures could be more sensitive to sleepiness than subjective measures^39^. Further studies including a sensitive objective measure may be required. Fourth, the measurements of distal skin temperature were performed for the dorsum sides of the feet and hands, according to a previous study^32^. It may be necessary to evaluate more suitable sites of the distal skin region that are involved in heat loss such as the plantar side. Hence, the association with the dorsum and plantar sides of the feet and hands should be investigated in a further study. Fifth, this study was conducted using a warming eye mask, which maintained the periocular skin temperature at 38–40°C for approximately 20 min. However, the duration of warming in the previous study was approximately 10 minutes^23^. Therefore, further investigation on applying the warming eye mask for varying durations will be necessary to clarify the relationship of warming duration and sleep.

In conclusion, periocular skin warming increased the distal skin temperature that is similar to physiological heat loss related with changes in body temperature before bedtime, leading to the acceleration of sleep onset and increase in delta power during the first half of sleep. These results suggest that skin warming manipulation in the periocular region may have favorable effects in promoting sleep onset by enhancing physiological heat loss.

## Methods

### Subjects

Twenty healthy volunteers recruited through a clinical research organization participated in this study. All subjects were free of any medical conditions and did not engaged in shift work. The detailed eligibility criteria and the sources of and methods of selection of subjects are described elsewhere^24^. All study procedures were approved by the Ethics Committee of Kao Corporation, and all subjects provided written informed consent after they received a detailed explanation of the experiment according to the Declaration of Helsinki. The study protocol was registered at the University Hospital Medical Information Network Clinical Trials Registry (UMIN-CTR registry ID: UMIN000029140) on September 14, 2017.

### Experimental protocol

We designed this study in a randomized placebo-controlled crossover manner. Subjects visited a laboratory for two experimental sessions that were 1 week apart. They were asked to keep their habitual sleep–wake schedule for 1 week prior to each session and to refrain from alcohol and caffeinated beverages for 24 h before each session. Subjects were randomly allocated to either a warming session or a sham session during the twice experimental sessions by an assistant staff in the clinical research organization. The study was conducted from October 16 to November 17 in 2017. The detailed experimental design is described elsewhere^24^.

**Figure 6:**
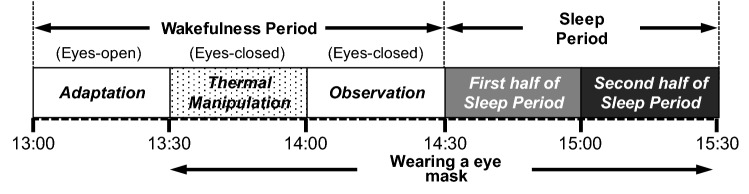
Schedule of the experimental session. In the thermal manipulation period, the periocular skin region temperature was manipulated by applying a warm or sham eye mask at 13:30. Subjects were instructed to maintain wakefulness with their eyes closed between the thermal manipulation and observation periods.

Figure 6 illustrates the schematic explanation in the experimental session. In each session, subjects were admitted to the laboratory by 10:30 and wore light experimental clothing. Then subjects stayed there until 11:30 while being allowed to read books or listen to music; however, they were prohibited from using cellular phones or watching digital monitors. At 11:30, a designated lunch (500 ~ 600 kcal) was provided to subjects, and the lunch was finished by 12:00. Then, the electrodes and thermistor probes were attached to subjects to obtain polysomnographic and body temperature recordings as described below. Subjects were instructed to keep a semi-recumbent posture on a bed with the head raised at approximately 35 degrees during the experimental period (13:00–15:30). The experimental period was divided into two periods; subjects were asked to remain awake during the first 90-min period (13:00–14:30) and try to sleep during the subsequent 60-min period (14:30–15:30). The 90-min wakefulness period consisted of three consecutive blocks, each with a duration of 30 min. In the first block (Adaptation period), subjects were required to maintain a semi-recumbent posture on a bed with their eyes open from 13:00 to 13:30. At 13:30, subjects were instructed to maintain wakefulness with their eyes closed until 14:30 and the periocular skin temperature was manipulated from 13:30 to 14:00 by placing a warming or sham eye mask on subjects’ periocular region during the block (Thermal manipulation period). In our prior study, the warming eye mask with active HSG sheets was confirmed to increase the periocular skin temperature gradually to 38°C within 5 min and ﻿maintain the skin temperature at 38–40°C for approximately 20 min, whereas the sham eye mask with inactive HSG sheets did not changed the skin temperature^24^. Changes in periocular skin temperature through the use of the warming and sham eye masks have been described in detail elsewhere^24^. Between 14:00 and 14:30, subjects were asked to maintain the same state as before, and the subjects’ physiological changes were observed in the final block (Observation period). Throughout these blocks, the subjects’ states were monitored visually and via EEG recordings by research assistants to enforce wakefulness, as described in detail elsewhere^24^. At 14:30, subjects were given a 60 min sleep period. Throughout these experimental periods, an ambient room temperature was maintained at 21–22°C and the light condition was maintained at dim light (~ 100 lx).

### Temperature recordings

Core body and skin temperatures were recorded using a portable device (LT-200, Gram Corp, Saitama, Japan) with a sampling rate of 1 Hz. The core body temperature was obtained using a thermistor that was self-inserted approximately 10 cm into the rectum. The skin temperature was measured at the following locations: the left and right sides of the feet (the middle of the dorsum side), hands (the middle of the dorsum side), infraclavicular regions, and left upper eyelids. The mean value of the left and right infraclavicular skin temperature was defined as the proximal skin temperature. The foot skin temperature of the left and right regions was averaged and was defined as the distal skin temperature. The distal–proximal skin temperature gradient (DPG) was calculated by subtracting the proximal skin temperature from the distal skin temperature. The detailed temperature recordings are described elsewhere^24^.

### Polysomnographic recordings

Polysomnographic (PSG) recordings were performed with the Biosignal Amplifier system (Polymate AP216, Miyuki Giken Corp., Tokyo, Japan) using four EEG channels (Fz, Cz, Pz, and O1, referenced against linked mastoid A1 and A2), two Electrooculogram (EOG)s channels (right and left outer canthus), a submental electromyogram (EMG), and electrocardiogram (ECG) channels. All data were sampled at 500 Hz. The heart rate was calculated from the R-R interval (RRI) detected on the ECG signal. The detailed polysomnographic recordings are described elsewhere^24^.

### Subjective sleepiness

Subjective sleepiness was evaluated using the Karolinska sleepiness scale^40^. Subjects were instructed to verbally reported their sleepiness at 0, 30, and 60 min after the eye mask placement. The subjects were pre-trained on how to verbally respond twice before application of the eye mask.

### Data analysis

Data on the core body temperature, skin temperature, DPG, and heart rate were respectively averaged over the 30-min thermal manipulation and observation periods, and the first and second halves of the 60-min sleep period. In order to examine the effects of thermal manipulation, the averaged core body temperature, skin temperature, DPG, and heart rate obtained during each period were subtracted by the respective baseline data taken just before the thermal manipulation period.

In accordance with the standard criteria^41^, the EEG recordings were divided into 30-s epochs, and classified into the following sleep stages using an automated stager (NightOwl Professional, NoruPro Light Systems, Inc., Tokyo, Japan)^42^: awake, rapid-eye-movement (REM), non-REM (NREM) sleep stage1, NREM stage2, and NREM stage3. The automatically staged data were verified by an investigator blinded to the conditions. SOL was defined as the time interval between 14:30 and the first onset of 3 continuous epochs of sleep defined by EEG.

Spectra analysis of EEG (Cz-A1) data was performed using a fast Fourier transform with a Hamming window. The power values for 5-s periods were obtained in the following four bands: delta (1.0–4.0 Hz), theta (4.0–7.0 Hz), alpha (8.0–12.0 Hz), and beta (13.0–20.0 Hz). The power activity in each band was normalized to the mean power value in each band across the sleep phase. Each power activity was averaged into the first half second halves of the sleep period. These analyses were performed with the software package MATLAB (The Math Works Inc., USA) and its signal processing toolbox.

All values are expressed as the mean ± standard error of the mean (SEM). All statistical analyses for comparisons between the sham and warming conditions were performed with the Wilcoxon signed-rank test. Correlations between possible two factors were analyzed using the Pearson’s product-moment correlation coefficient. Probability values of less than 0.05 were accepted as statistically significant. All statistical analyses were performed using IBM SPSS Statistics version 25 (IBM Corp., Chicago, IL, USA).

## Data Availability

The datasets analyzed during the current study are available from the corresponding author on reasonable request.
